# Increased persistence of large-scale circulation regimes over Asia in the era of amplified Arctic warming, past and future

**DOI:** 10.1038/s41598-020-71945-4

**Published:** 2020-09-11

**Authors:** Jennifer A. Francis, Natasa Skific, Stephen J. Vavrus

**Affiliations:** 1 Woodwell Climate Research Center, Falmouth, MA USA; 2 Department of Marine and Coastal Sciences, Rutgers University, New Brunswick, NJ USA; 3 Nelson Institute Center for Climatic Research, University of Wisconsin-Madison, Madison, WI USA

**Keywords:** Atmospheric science, Climate change, Cryospheric science

## Abstract

Extreme weather events in Asia have been occurring with increasing frequency as the globe warms in response to rising concentrations of greenhouse gases. Many of these events arise from weather regimes that persist over a region for days or even weeks, resulting in disruptive heatwaves, droughts, flooding, snowfalls, and cold spells. We investigate changes in the persistence of large-scale weather systems through a pattern-recognition approach based on daily 500 hPa geopotential height anomalies over the Asian continent. By tracking consecutive days that the atmosphere resides in a particular pattern, we identify long-duration events (LDEs), defined as lasting longer than three days, and measure their frequency of occurrence over time in each pattern. We find that regimes featuring positive height anomalies in high latitudes are occurring more often as the Arctic warms faster than mid-latitudes, both in the recent past and in model projections for the twenty-first century assuming unabated greenhouse gas emissions. The increased dominance of these patterns corresponds to a higher likelihood of LDEs, suggesting that persistent weather conditions will occur more frequently. By mapping observed temperature and precipitation extremes onto each atmospheric regime, we gain insight into the types of disruptive weather events that will become more prevalent as particular patterns become more common.

## Introduction

A new hypothesis linking observed rapid Arctic warming and melting during recent decades with an increased likelihood of extreme weather events in midlatitudes was proposed^1^. The basic idea was that disproportionate Arctic warming, mainly owing to a variety of positive feedbacks involving the disappearance of ice and snow^2,3^, is reducing the north/south temperature gradient, which is the primary driver of the polar jet stream. Observations indicate that when the westerlies of the jet stream are weaker, a wavier jet-stream path is favored (e.g., see Fig. 11 in^4^), and large waves tend to progress eastward more slowly. The dynamics of these planetary waves generate weather regimes of all sorts, thus when they move slowly, weather conditions persist and can cause extreme weather events.

Since this controversial study, many dozens of papers have been published investigating various parts of this hypothesis. The vast majority of studies based on reanalysis data support an association between amplified Arctic warming (AAW) and changes in the large-scale circulation that are consistent with slower-moving planetary waves, while investigations using model simulations are split as to whether a robust connection exists^5^. What is abundantly clear, however, is that challenges remain in identifying cause-and-effect because the signal-to-noise ratio is small, many changes are happening simultaneously in the climate system, models tend to underestimate the atmospheric response^6,7^, and model physics critical to the linkage may be unrealistic or missing^8^. Meanwhile, the controversy about the Arctic’s role in changing midlatitude weather continues, as some new studies report little atmospheric response to AAW^9,10^ while others identify a robust response^5^.

Here we focus on the last piece of the hypothesis—the changing persistence of large-scale weather patterns—which has received relatively little attention. Existing studies of weather persistence tend to focus on duration of temperatures over or under some threshold, or number of days with or without precipitation. A few recent studies provide evidence of changing weather persistence in the northern hemisphere. For example, one study^11^ found that precipitation occurring on two or more consecutive days at weather stations in the northeastern U.S. increased in all months during the past several decades, particularly in late spring. Positive trends were also found in spring dry spells, implying more variable precipitation regimes. Also using precipitation measurements from nearby (~ 50 km) groups of weather stations, another study^12^ expanded the domain to span the U.S. and found that the frequency of long-duration wet and dry spells (three or more consecutive days) has changed, especially in the past few decades. Wet spells generally have become more frequent over some areas of the east in all seasons, while central and southeastern regions have experienced more frequent dry spells.

Others researchers^13^ investigated the persistence of temperature anomalies using 60 years of station data on northern hemisphere continents. They analyzed changes in duration of unusually warm or cold days using a pattern-recognition approach to identify areas that exhibit similar behavior. Their results indicate that both warm and cold anomalies had become more persistent during summer months, particularly heat waves in Europe, and that long-duration events were more likely to occur when storm tracks were weak owing to smaller poleward temperature gradients. This work was extended using projections from an atmosphere-only climate model^14^. Under conditions of 2 °C global warming, they found that persistent (longer than two weeks) warm anomalies during summer increased in frequency by 4%, while long-duration precipitation events (longer than 7 days) increased by 26%. The combination of both warm and dry conditions during summer became 20% more likely in eastern North America.

Assessing weather-regime persistence using near-surface temperatures measurements, however, is problematic because at a particular location, the temperature can be affected by factors other than the large-scale weather regime. These factors may include winds blowing from dissimilar surfaces (such as bodies of water or bare soil), topographic effects (downslope or upslope winds), or passing areas of cloud cover. This study^15^ focused on persistence in an approximately 1,000 km^2^ region of Europe using daily 500 hPa wind directions from model simulations averaged over the area. While they find increasingly dry and warm conditions occurring during summer, they find no robust signal of increased persistence. The same mean wind direction over a region of this size, however, could result from differing patterns of 500 hPa heights, thus the persistence of the overarching pattern may be inconsistent with their results.

Regional-scale precipitation holds more promise as an indicator, as conditions that are conducive for precipitation are generally dictated by large-scale rather than local features (except for isolated airmass convection). Even more promising is an approach based on large-scale circulation regimes. These studies^12,16^ employed a pattern clustering tool called self-organizing maps (SOMs) to investigate changes in persistence of large-scale weather-regimes. They analyzed representative patterns in 500-hPa geopotential height anomalies over the North Atlantic/Europe^16^ and the eastern Pacific/North America^12^ to measure the frequency of long-duration events (LDEs), defined as cases when the atmosphere resides in one pattern for four or more consecutive days. Both studies found that LDEs increased (decreased) during the period from 1996 to 2015 relative to 1976 to 1995 in patterns with positive (negative) height anomalies in high latitudes. Over Europe, the regimes with more (less) frequent LDEs resemble negative (positive) North Atlantic Oscillation conditions, which are typically associated with cold (warm) winters. These results based on the persistence of large-scale regimes suggest that both continents will experience more extreme weather events associated with long-duration conditions.

Adding to this body of evidence, we build on this work^12^ by assessing the persistence of large-scale atmospheric patterns over the Asian continent during the past, with reanalysis output and historical model simulations, and into the future with model projections. We find that patterns featuring warm (cold) high-latitudes generally exhibited increased (decreased) frequencies of LDEs in recent decades, and historical simulations from the four coupled global models we analyzed were able to capture similar behavior. Simulations for the future, assuming business-as-usual conditions, project substantially larger increases (decreases) in the frequency of LDEs for patterns featuring warm (cold) Arctic conditions.

## Data and methods

We identify representative large-scale atmospheric patterns over Asia using a neural-network-based tool called self-organizing maps (SOMs)^17^. The SOM algorithm ingests large, two-dimensional data sets and groups the fields into clusters or nodes of representative patterns found in the data. The patterns are arranged in a variable-size matrix according to their similarity with each other, with most similar patterns positioned near each other and most dissimilar patterns farthest apart. For this application we use daily fields of anomalies in the 500-hPa geopotential heights from 1948–2018 (~ 26,000 days) obtained from the National Center for Environmental Prediction/National Center for Atmospheric Research (NCEP/NCAR) Reanalysis^18^. The spatial domain extends from 30^o^N-80^o^N and 30^o^E-180°, and daily anomalies were calculated by subtracting the 71-year mean value for each gridpoint for that calendar day. We note that 500 hPa height fields from other reanalyses are very similar^19^.

For this application, we chose a 4 × 3 SOM matrix, which balances sufficient representation of the atmosphere’s dominant patterns with ease of displaying of results. The algorithm places each daily field into the node most similar to data on that day. Once this so-called master SOM has been created, other fields of data can be mapped to the patterns, which is especially powerful if the other variables are heterogeneous or poorly behaved (e.g., cloud cover, radiation, or extreme events). We take advantage of this tool to explore extreme temperatures and precipitation associated with each pattern. Changing frequencies of occurrence of the SOM patterns allow an assessment of trends in extremes. Temperature and precipitation data are also obtained from the NCEP/NCAR Reanalysis. While precipitation data are notoriously error-prone in terms of absolute magnitude, mean values at the grid-box scale (2.5°) should reasonably capture patterns and changes over time. Precipitation fields in the NCEP reanalysis compared favorably with values from the global precipitation climatology project (GPCP), especially in terms of large-scale variability and interannual variability^20^.

Output from models participating in the Climate Model Intercomparison Project version 5 (CMIP5) are also analyzed, both from historical simulations (1979–2005) and Representative Concentration Pathway 8.5 (RCP8.5) projections for 2006–2100. We accessed simulations of daily 500 hPa height fields from the following three models: NCAR’s Community Climate System Model version 4 (CCSM4), Canadian Earth System model version 2 (CanESM2), and Geophysical Fluid Dynamics Laboratory Climate Model Version 3 (GFDL-CM3). All data were obtained from https://esgf-node.llnl.gov/search/cmip5/. Historical runs incorporate observed natural and anthropogenic forcings, while future projections assume conditions defined by the RCP8.5 scenario^21^. Anomalies in future projections were calculated relative to the mean from 2006 to 2100

Following the approach used in previous investigations^12,16^, persistence in this study is assessed by identifying long-duration events (LDEs), defined as cases when the large-scale atmospheric pattern remains static for four or more consecutive days. The size of the matrix was selected to be relatively small, thus ensuring that adjacent patterns were distinct from each other and so consecutive days would be less apt to jump between adjacent patterns that differed little. Because the SOM algorithm tracks all days that belong in each node, the frequency, duration, and changes in LDEs over time can be tracked by identifying strings of consecutive days in each node. LDEs can then be associated with other atmospheric fields, including anomalies in temperatures and precipitation. Additional details on this analysis method are available^12^.

## Results

### The Master SOM

**Figure 1:**
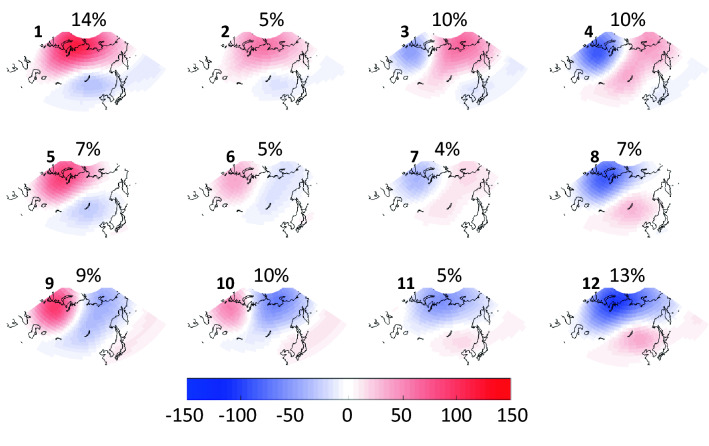
Matrix of representative patterns in 500-hPa geopotential height anomalies created using the self-organizing map algorithm. Daily data from the NCEP/NCAR reanalysis span 1948 to 2018, 30°N to 80°N, and 30°E to 180° longitude. Shading displays height anomalies in meters, and percentages over each pattern denote the occurrence of days in that node. Large bold numbers are for node reference.

The SOM algorithm identified the representative patterns of 500 hPa geopotential height anomalies displayed in Fig. 1. Patterns in diagonal corners tend to display nearly opposite features. The percentage of days belonging in each node is displayed above each plot. Central nodes are the least frequent while corner patterns tend to occur most often.

Nodes in the upper left section of the SOM exhibit positive (negative) height anomalies in high (middle) latitudes, indicative of warm Arctic conditions accompanied by cool anomalies particularly in East Asia. Nodes in the lower right display negative height anomalies in high latitudes along with high heights over East Asia. Anomalous wind fields can be inferred from height patterns. In nodes #1 and #5, for example, anomalous northeast winds would be expected in central Asia. Cold Arctic winds from the north blow through central Asia in #9 and #10, while warm southerlies would be expected in #3 and #4.

**Figure 2:**
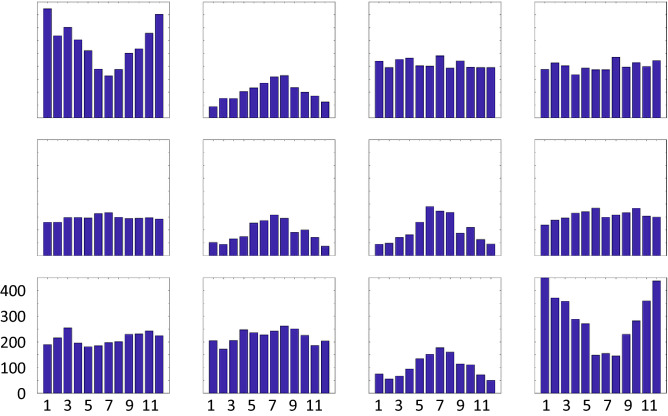
Monthly distribution of days residing in each node of the master SOM (Fig. 1). Units are months (x-axis) and days (y-axis), indicated on bottom left node.

We can also assess the seasonal occurrence of each pattern based on the days that reside in it. Figure 2 illustrates that patterns #1 and #12, which exhibit the most extreme opposite features, occur most often during cold months, while patterns with weak features (#2, #6, #7, and #11) are more likely in the warm season. This result is consistent with observations of strong (weak) baroclinicity and large airmass differences during winter (summer). Four of the patterns occur nearly equally in all months.

### Extreme temperatures and precipitation

**Figure 3:**
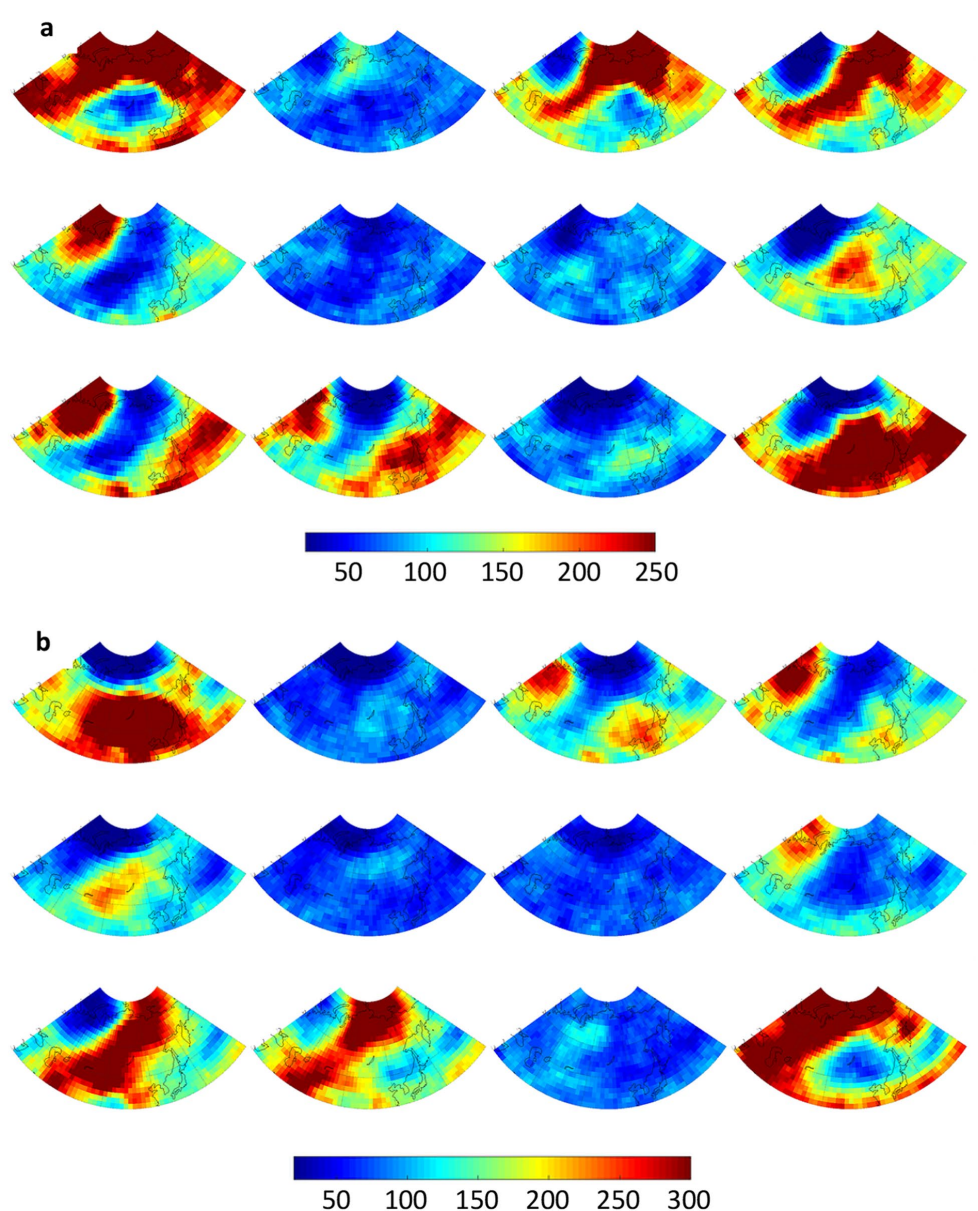

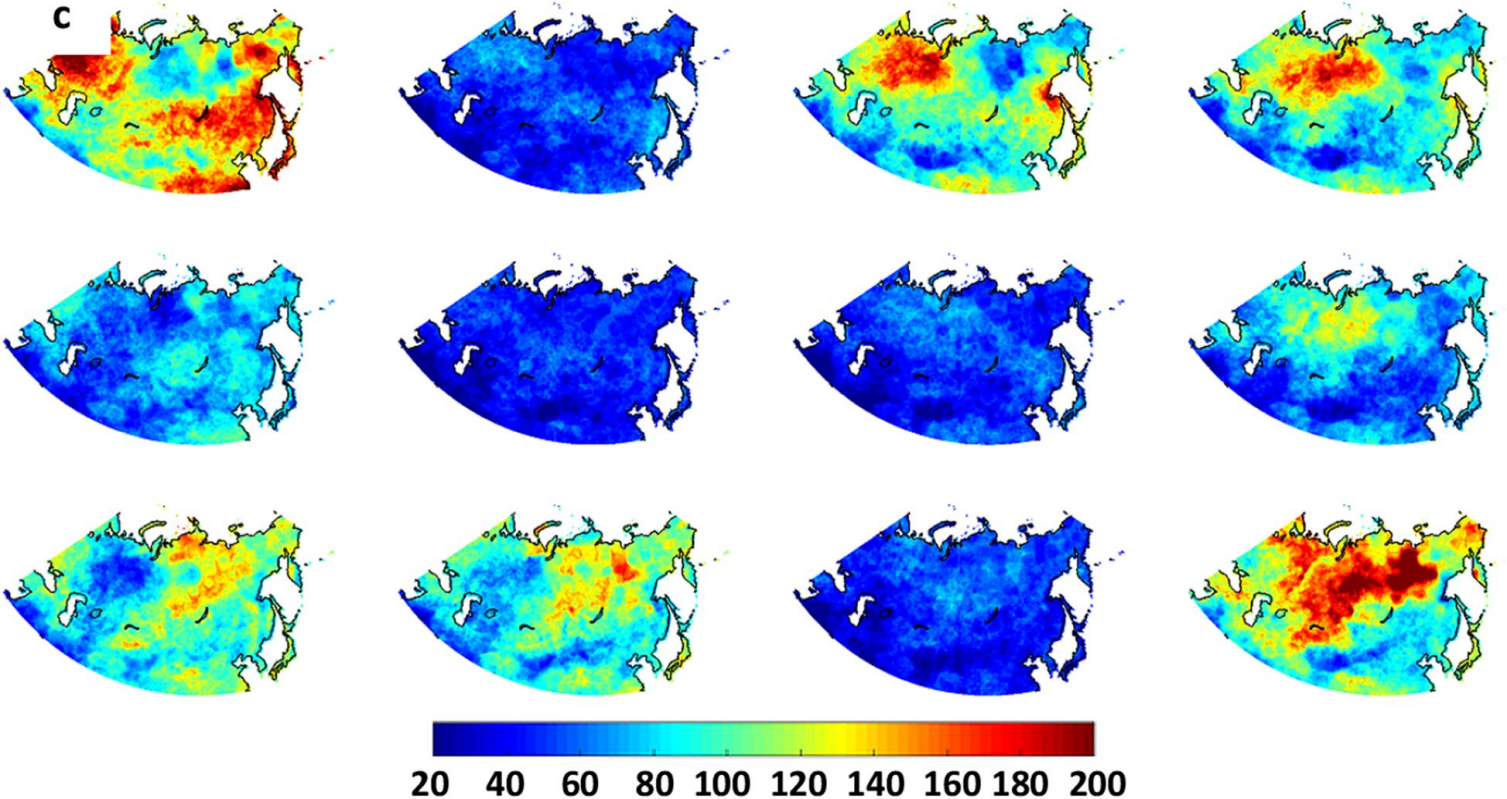
Number of days that (**a**) 925 hPa air temperatures exceed 1.5 σ, (**b**) 925 hPa air temperatures fall below -1.5 σ, and (**c**) total precipitation exceeds 1.5 σ.

Next we investigate the frequency and spatial distribution of extreme weather conditions associated with each large-scale pattern. Anomalies in air temperatures at 925 hPa (chosen to avoid small-scale surface effects) and total precipitation (relative to mean over entire record) are calculated from daily reanalysis output. The number of extreme days occurring at a gridpoint—i.e., days exhibiting anomalies above or below 1.5 σ—are mapped to the matrix and displayed in Fig. 3. Areas with a high frequency of extremely warm days (Fig. 3a) correspond fairly closely with positive height anomalies in the master SOM (Fig. 1), and also with regions where anomalous winds with a southerly component would be expected based on the location and orientation of height anomalies. A few interesting cases are evident, such as the large region of frequent warm extremes in node #12 across most of central and eastern Asia as well as the western Pacific, even though positive height anomalies are weak in those areas. Extremely high temperatures in these regions correspond with low heights in the Arctic. The same is true for #10, where extreme warmth is common across southeastern Asia where height anomalies are nearly neutral but low heights exist in the Siberian Arctic. Also noteworthy is that the most widespread warm extremes are associated with patterns that exhibit strong and meridionally oriented height anomalies, not the weak patterns that occur most often in summer months (Fig. 2).

Similarly, cold temperature extremes (925 hPa air temperatures < − 1.5 σ) are mapped to the SOM in Fig. 3b. While patterns look similar to but opposite those in Fig. 3a, interesting exceptions are evident. A large region of frequent cold extremes spans much of Asia in node #1, a pattern characterized by an anomalously warm Arctic (positive height anomalies) and only weakly negative values over eastern Asia. Nodes #9 and #10 also exhibit regions of high heights in central and western Siberia, along with a broad band of cold extremes extending from southwestern Asia to northeastern Siberia. Interestingly, height anomalies in node #5 look similar but cold extremes are absent, likely because the height gradient is weaker and has a more zonal orientation.

A similar analysis is performed using extreme precipitation values, i.e., exceeding 1.5 σ (Fig. 3c). Widespread precipitation extremes are associated with nodes #1 and #12, which are most prevalent in the cold months (Fig. 2). In the “warm Arctic” pattern of node #1, days with heavy precipitation occur frequently over northwest and southeast Asia, as well as along the east coast. The “cold Arctic” node #12 pattern is associated with precipitation extremes across much of northern Asia, extending southward into western Asia. Nodes #3 and #4 feature anomalous flow from the south into northwest/northcentral Asia, producing heavy precipitation there, while nodes #9 and #10 tend to bring extreme precipitation into central and eastern regions associated with anomalous flow around the negative height anomaly in the northeast part of the continent. The nodes with maximum occurrence during summer months (#2, #6, #7, and #11) exhibit a small number of days with extremes in either temperatures and precipitation.

**Figure 4:**
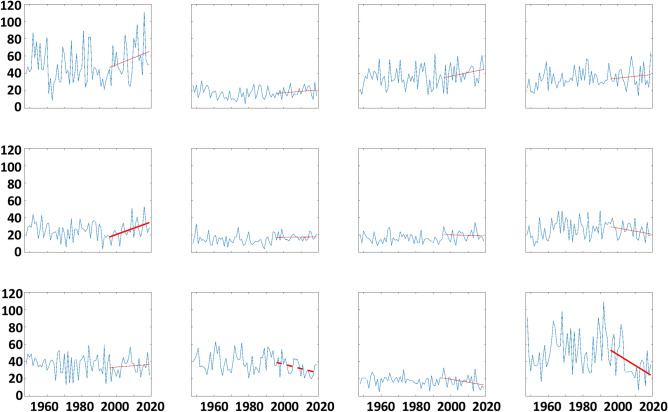
Change in the frequency of occurrence of each node from 1948 to 2018; units in days per year. Dashed (solid) lines indicate trends significant at the 90th (95th) confidence level from 1996 to 2018.

Societally important questions receiving a great deal of attention recently are how and why extreme weather conditions have changed as the globe has warmed, and what does the future hold? We can shed light on this issue through SOM analysis by assessing changes in the frequency of large-scale atmospheric patterns over time, and relating them to the extreme conditions associated with those patterns. Figure 4 presents time series of days/year that belong in each node of the SOM. Trends significant at 90% (95%) confidence are indicated with dashed (solid) bold lines, determined with the f-test. Nodes that exhibit positive height anomalies in high latitudes are generally occurring more frequently over time, especially since 1995 coincident with rapid Arctic warming. This is particularly evident in node #5 (> 95% confidence), with increases of fairly high confidence in node #1 (> 85%). Patterns with negative height anomalies in high latitudes, in contrast, are generally decreasing, especially in recent decades. This finding is consistent with observations of amplified Arctic warming^2,22^.

These trends in particular patterns help explain the recent increased heat extremes in the Arctic, western Asia, and eastern Asia (Fig. 3a, nodes #1, #3, and #4), as well as more frequent extreme cold days in central and eastern Asia (Fig. 3b, nodes #1 and #3) and decreased cold events in western areas (nodes #10 and #12). Changes in pattern frequency also contribute to increased days with heavy precipitation over western, southcentral, and eastern Asia (Fig. 3c, nodes #1, #3, and #4) along with decreased precipitation extremes over northern Asia (node #12).

### Persistence assessed via frequency of long-duration events (LDEs)

**Figure 5:**
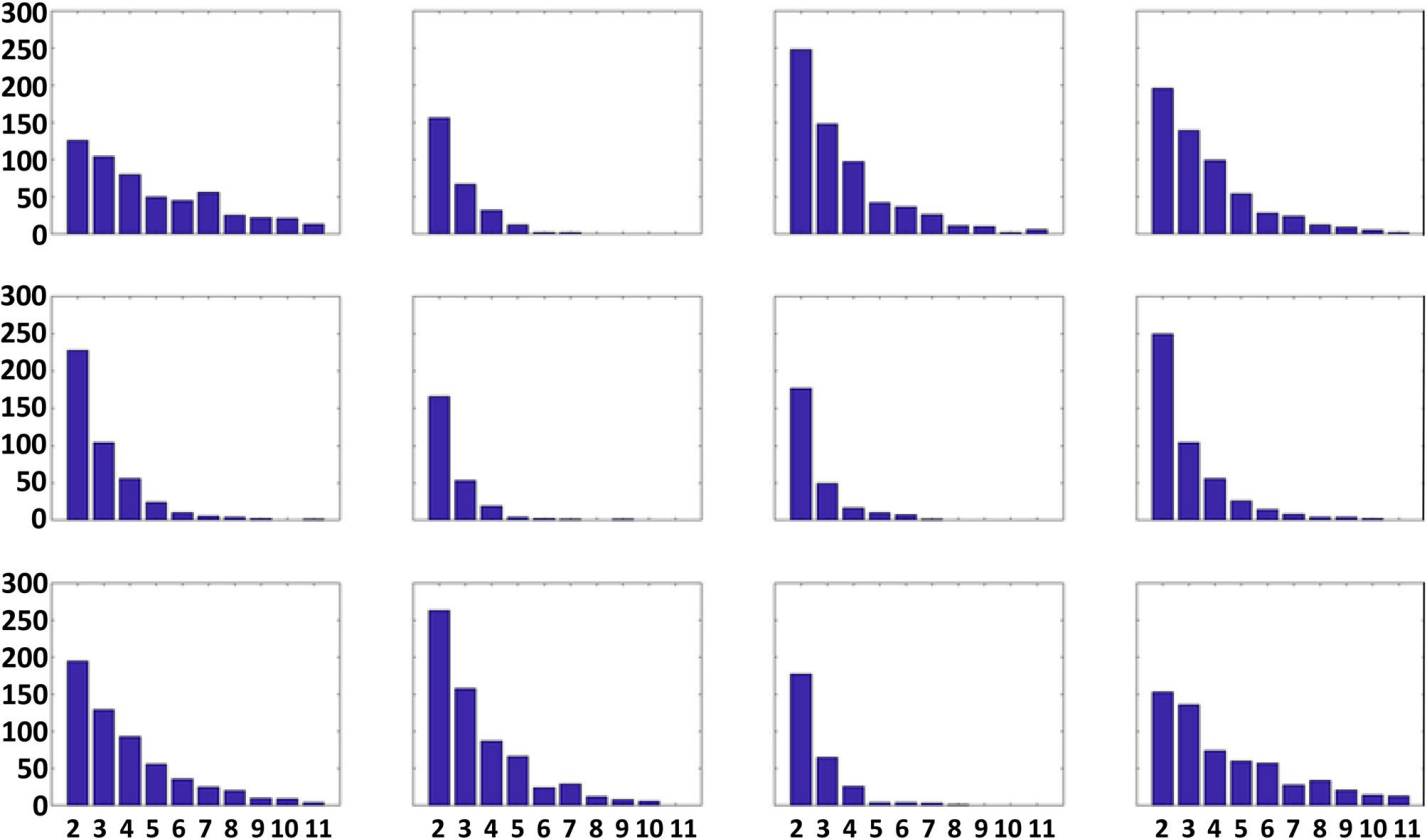
Distribution of LDEs of varying duration in each node. The horizontal axis is length of LDEs in days, and the vertical axis is total number of LDEs of each duration.

As described above, we identify LDEs by tracking events in which four or more consecutive days occur in each node of the SOM. This threshold was determined subjectively by assessing the distribution of LDEs of varying lengths, as illustrated in Fig. 5. As expected, long-duration LDEs occur less frequently than short ones, with a dearth of events longer than three or more days duration. This threshold was selected to provide sufficient cases to detect statistically significant changes over time.

**Figure 6:**
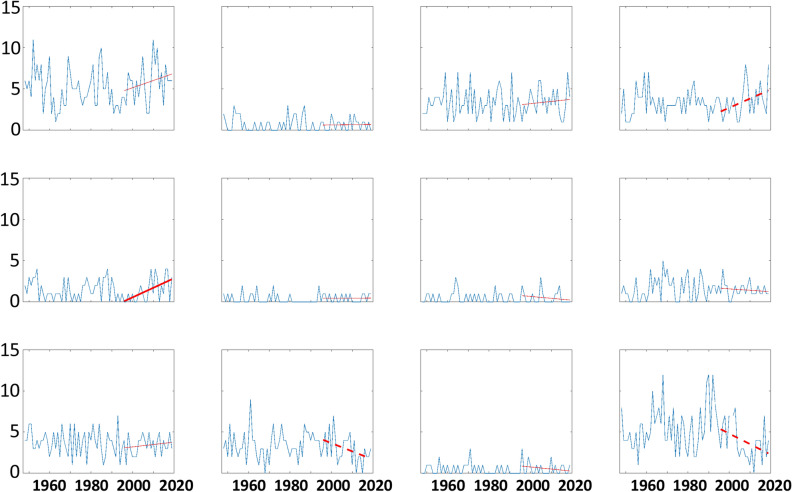
Timeseries of the occurrence of LDEs (defined as 4 or more consecutive days in a node) per year based on NCEP/NCAR Reanalysis output. Bold dashed (solid) lines indicate trends from 1996-2018 significant at the 90th (95th) confidence level.

**Figure 7:**
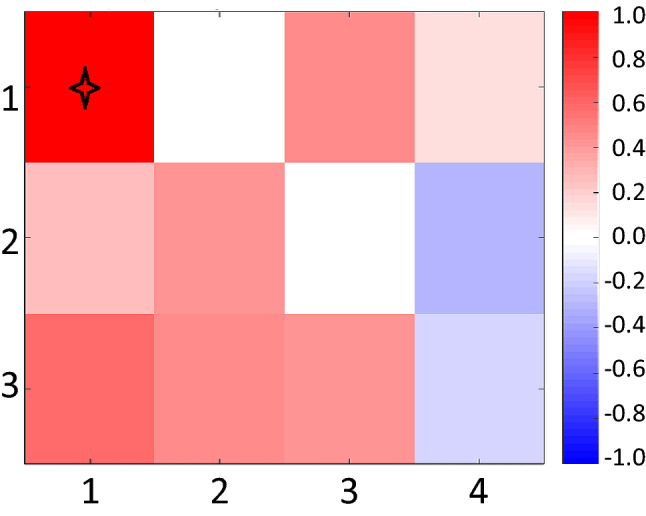
Change in the probability (%) of LDE occurrence (number of LDEs divided by number of days in a node) from 1962-1989 to 1991-2018 for LDEs lasting 7 or more days. Star indicates significance at 95% confidence.

Time series of LDEs ≥ 4 days per year from 1948 to 2018 for each node are presented in Fig. 6. Large interannual variability is evident, and there are no significant trends over the 71-year period, although significant positive (negative) trends are evident in nodes #4 and #5 (#10 and #12) since the mid-1990s. It appears that none of these recent trends are consistently associated with changes in occurrence of short-duration (2 or 3 consecutive days) events (Fig. S1). We also find that the relative probability of an LDE occurring—especially longer LDEs—has increased significantly in recent decades for node #1 (Fig. 7; warm Arctic pattern), thus the change in frequency of LDEs results from the combined effects of the atmospheric pattern occurring more frequently (thus increasing the chance of multiple consecutive days) plus an increased probability of an LDE occurring per day.

### Analysis of model simulations

Once the master SOM has been created, fields from other sources can be mapped onto the same master patterns. We take advantage of this analysis tool by mapping output from climate model simulations to the SOM matrix in Fig. 1. The algorithm places daily fields of 500 hPa height anomalies from each of three models into the node whose pattern is most similar to the modeled field. By using the master patterns identified in reanalysis output, any unrealistic patterns (or unrealistic dominance of any pattern) that may exist in model simulations are avoided.

We first compare historical model output with reanalysis results to assess the models’ ability to reproduce monthly distributions of node frequencies (Fig. S2). All three models do a remarkable job of capturing the frequency of patterns and their seasonal distribution. The frequencies and temporal changes in LDEs are also broadly similar (Fig. S3), with generally decreasing frequencies of patterns featuring negative height anomalies at high latitudes, though internal variability clearly dominates over this relatively short time period (1979–2005).

Having demonstrated that model simulations are able to capture realistic distributions of node occurrence and frequencies of LDEs in each node, we now turn to projections for 2006 to 2100 assuming future conditions described by the RCP 8.5 scenario. We further assess the realism of model output by creating two additional entirely independent SOM matrices using historical simulations (not shown) and future projections from the CCSM4 model (Fig. S4). Patterns in this matrix are nearly identical to those created using reanalysis fields (Fig. 1), and the monthly distributions are also very similar. These results provide further confidence in the model’s realism and suggest that the representative atmospheric patterns will not fundamentally change in the future nor shift in their monthly distributions.

**Figure 8:**
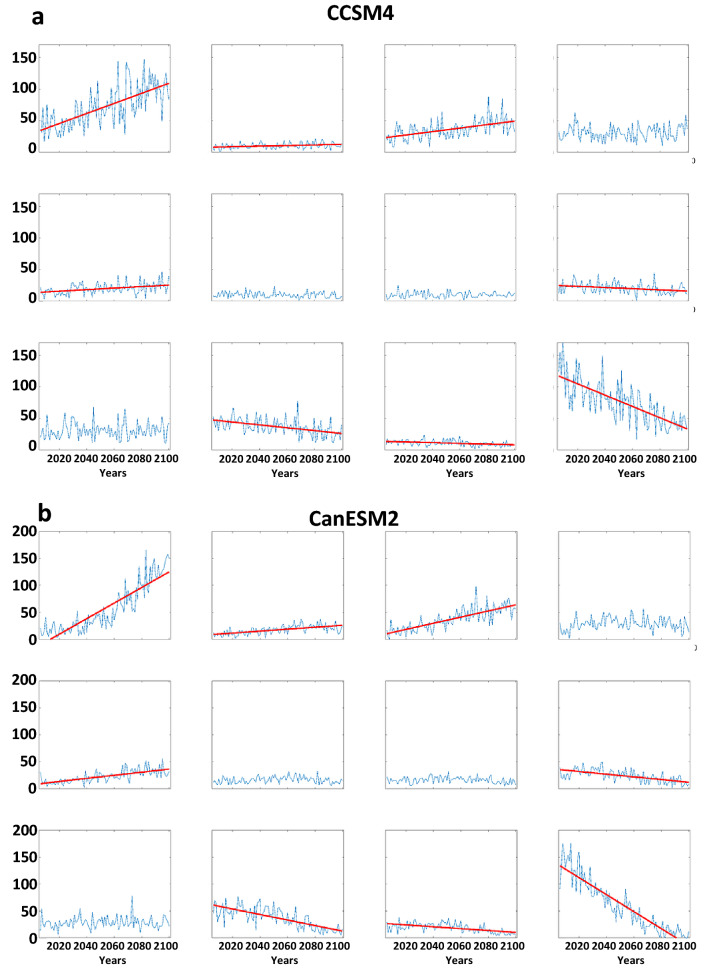

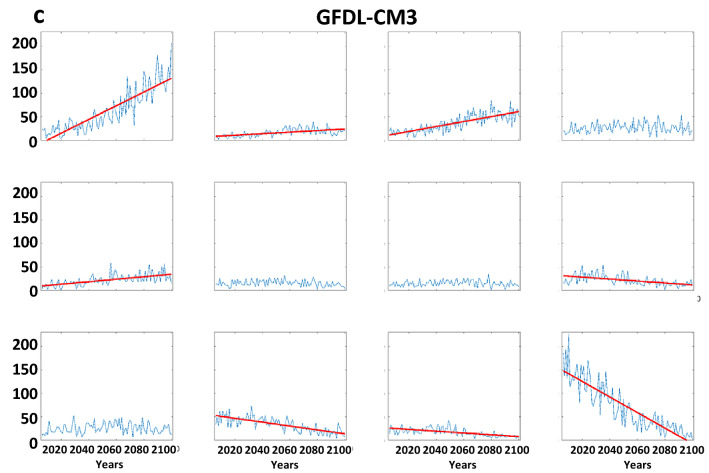
Model projections of node frequencies from 2006 to 2100 by the (*a*) CCSM4, (**b**) Can-ESM2, and (**c**) GFDL-CM3. Simulations are for the RCP 8.5 scenario. Dashed (solid) lines indicate significant trends with 90% (95%) confidence.

Time series for the days/year in each node are presented in Fig. 8. Trends apparently emerging in the recent past (Fig. 4) are much more conspicuous in future projections. Generally, nodes featuring positive height anomalies in high latitudes (upper-left nodes in master SOM, Fig. 1) occur much more frequently in the most aggressive warming scenario of the CMIP5 experiment. From 2006 to 2100, the number of days residing in node #1 is projected to increase by about 150% in CCSM4 simulations, with even larger increases projected by CanESM2 (~ 600%) and GFDL-CM3 (~ 650%). This finding is consistent with expected intensification of disproportionate Arctic warming as greenhouse gases continue to accumulate in the atmosphere^23^. The AAW-like patterns increase at the expense of those with low height anomalies in the Arctic; in fact, these patterns become exceedingly rare by the end of the twenty-first century in all three models.

In terms of extreme weather, assuming the patterns of anomalous temperatures and precipitation associated with each node hold for the future, the projected trends in pattern frequency should have a substantial impact. Many more days residing in node #1, for example, should contribute to an increased frequency of warm extremes in western, northern, and eastern Asia; more extreme cold days in central and eastern Asia; and heavy precipitation in western and southeastern areas. Many fewer days in node #12, in contrast, will favor a decrease in warm extremes over southern Asia, fewer cold days in western and northern regions of the continent, and fewer precipitation extremes in northern and southwestern regions.

**Figure 9:**
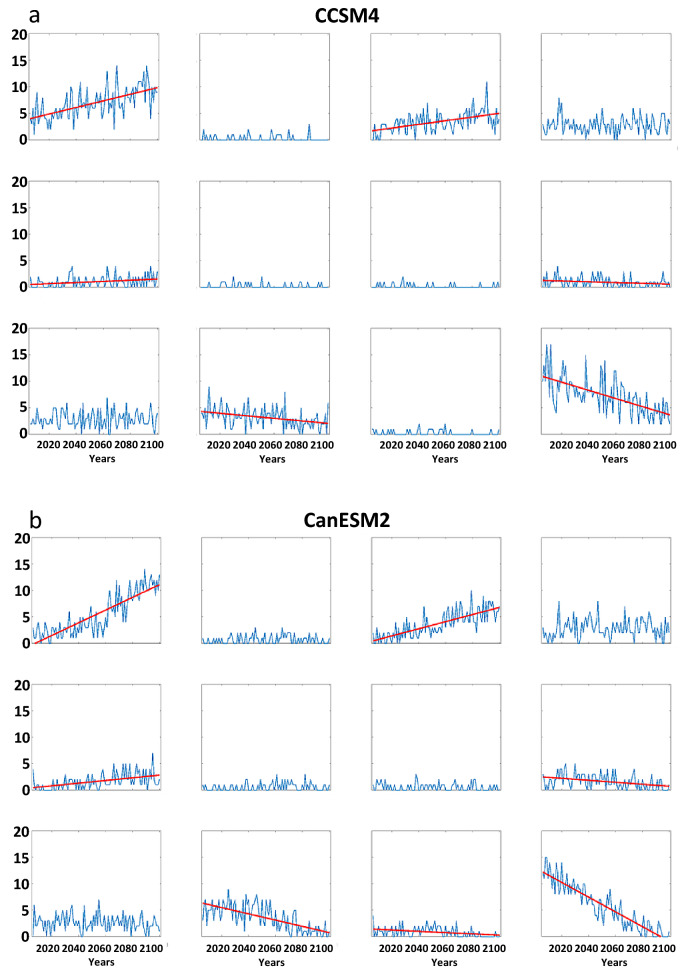

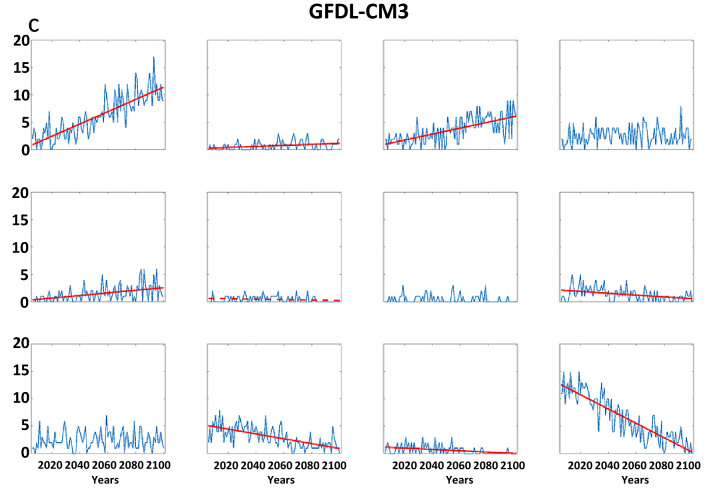
Model projections of LDE frequencies per year from 2006 to 2100 by the (**a**) CCSM4, (**b**) Can-ESM2, and (**c**) GFDL-CM3. Simulations are for the RCP 8.5 scenario. Dashed (solid) lines indicate significant trends with 90% (95%) confidence.

Trends in the frequency of LDEs generally follow the changes in node occurrence (Fig. 9). This is to be expected, as a larger number of days belonging in a node will increase the chances of events with multiple consecutive days. As in the analysis based on observations, we find that the probability-per-day of long LDEs generally increases in AAW patterns derived from future projections, as well (Fig. S5). The combination of these changes suggests that the future will bring more frequent occurrences of persistent weather patterns. It should be noted that no negative trends (in node frequency or LDEs) occur for patterns with positive high-latitude height anomalies, and no positive trends occur in nodes with a cold Arctic. Even in the more optimistic RCP scenarios, disproportionate Arctic warming is expected to continue, and these results suggest that it will be accompanied by more frequent persistent weather patterns as well as the specific types of extreme events across the Asian continent associated with those patterns.

## Discussion and conclusions

In this study we have addressed the hypothesis that amplified Arctic warming will contribute to an increased frequency of persistent weather patterns over Asia, which will in turn lead to more frequent occurrence of certain extreme weather events. Our approach was to employ an algorithm to identify characteristic large-scale atmospheric patterns over the region using reanalysis output, analyze spatial patterns of extreme temperatures and precipitation associated with those patterns, then assess how each circulation regime has changed in frequency. Further, we repeated the analysis using historical simulations and future projections from three coupled climate models participating in the CMIP5 experiment forced with RCP 8.5 conditions.

Focusing on large-scale patterns rather than station data has some distinct advantages. Local geographic features can exert a strong influence on weather conditions, and depending on the heterogeneity of the area surrounding a location, an observation may represent only a small area. The regime approach used in this study eliminates this issue and can also be more easily linked with teleconnection indices, wind anomalies, energy transport, synoptic weather systems, and regional weather conditions.

Our findings are largely consistent with previous studies that reported changes in extreme weather events and regime persistence for the Asian continent. For example, this study^24^ used an atmospheric GCM forced with reduced Arctic sea ice (a proxy for amplified Arctic warming consistent with node #1) to investigate changes in the frequency and duration of cold and warm spells as well as precipitation extremes. Over Siberia they found a significant increase in the frequency and duration of warm spells and wet days, while central Asia saw more cold spells and wet days, and east Asia experienced more long wet spells. These results are consistent with an increased (decreased) prevalence of the pattern in node #1 (#12). They also found that warm, wet, and dry spells predominantly lengthened in most parts of Asia, suggesting a general increase in persistence. Another study^14^ analyzed output from several atmosphere-only models forced by sea-ice and ocean-temperature conditions consistent with a 2 °C warmer world. Similar to our results, they found significantly increased persistence of warm spells over northern and central Asia, as well as wet spells over northern and eastern Asia.

In addition to supporting previous findings, our study demonstrates an increasing frequency of persistent large-scale circulation regimes and associated extreme weather events, especially since the mid-1990s when AAW emerged as a clear signal. As greenhouse gases continue to accumulate in the atmosphere owing to ongoing human activities, we find that patterns characterized by warming in high latitudes will occur more frequently while cold-Arctic patterns will decline. A higher percentage of days/year in any one pattern will increase the likelihood of multiple consecutive days occurring in that pattern, leading to more frequent persistent conditions. Moreover, we demonstrate that the predominant warm-Arctic pattern also exhibits a higher probability of long LDEs occurring relative to days belonging in a node, thus further augmenting the likelihood of persistent weather events. Three of the climate models participating in CMIP5 agree that warm-Arctic patterns will increase several-fold by the end of the century at the expense of cold-Arctic patterns, suggesting a substantial rise in the frequency of persistent circulation regimes and their associated extreme weather. The connections with changes in jet-stream characteristics, such as blocking and other cut-off circulation features, will be addressed in future work.

## Supplementary information

Supplementary file1
