# Supplementary material for: Increased persistence of large-scale circulation regimes over Asia in the era of amplified Arctic warming, past and future
Source: Sci Rep. 2020 Sep 11;10:14953. doi: 10.1038/s41598-020-71945-4 (PMC7486397; doi:10.1038/s41598-020-71945-4)
Supplement: Supplementary file 1 — Supplementary file1 [file 41598_2020_71945_MOESM1_ESM.docx]

# *Supplemental Material*

# Increased persistence of large-scale circulation regimes over Asia in the era of amplified Arctic warming, past and future

Jennifer A. Francis*, Woodwell Climate Research Center, Falmouth MA, USA, *jfrancis@woodwellclimate.org*

Natasa Skific, Dept. of Marine and Coastal Sciences, Rutgers University, New Brunswick, NJ, *natasa.skific@verizon.net*

Stephen J. Vavrus, Nelson Institute Center for Climatic Research, University of Wisconsin-Madison, WI, *sjvavrus@wisc.edu*

Revised version submitted to *Nature Scientific Reports*, 1 September 2020

*Corresponding author

# Supplemental Material


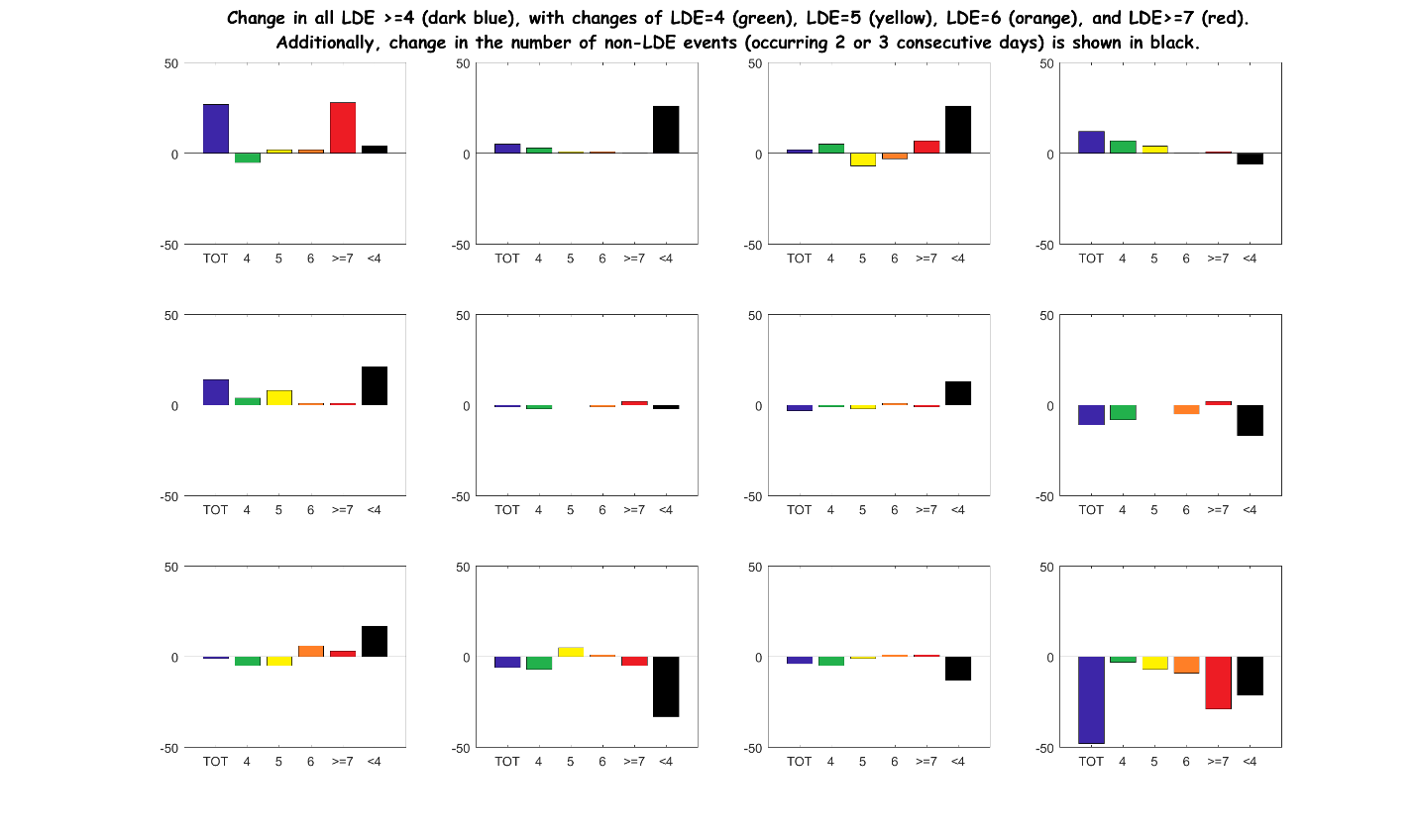


**Figure S1:** Changes in frequency of consecutive-day events from 1962-1989 to 1991-2018. Blue is for all LDEs (≥ 4 consecutive days), green is for 4 days, yellow is for 5 days, orange is for 6 days, red is for ≥ 7 days, and black is for non-LDEs (2 or 3 consecutive days).


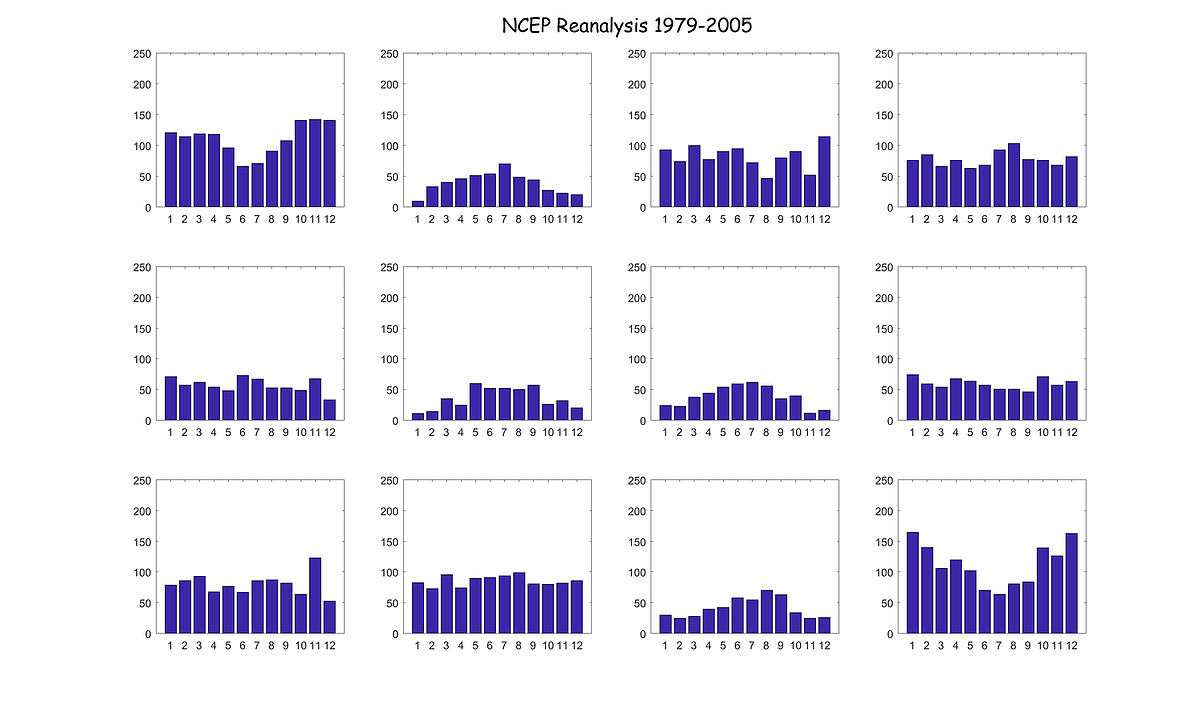

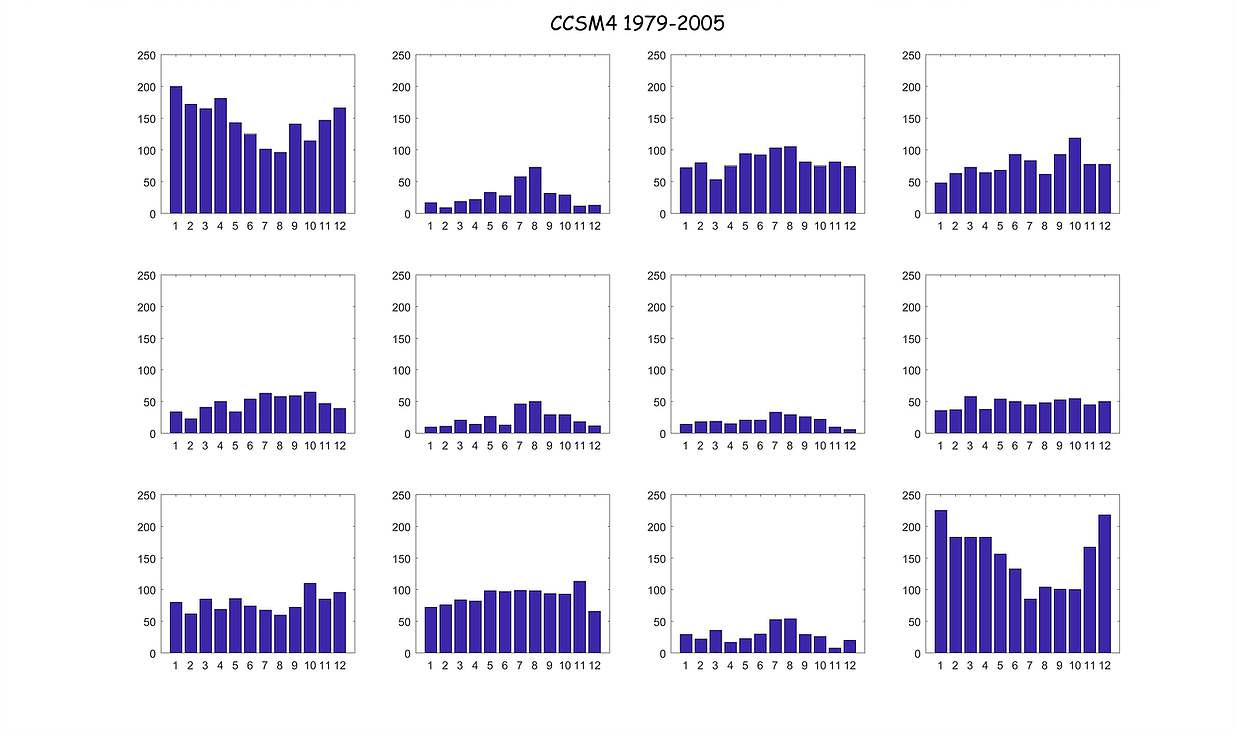


**d**

**c**

**b**

**a**


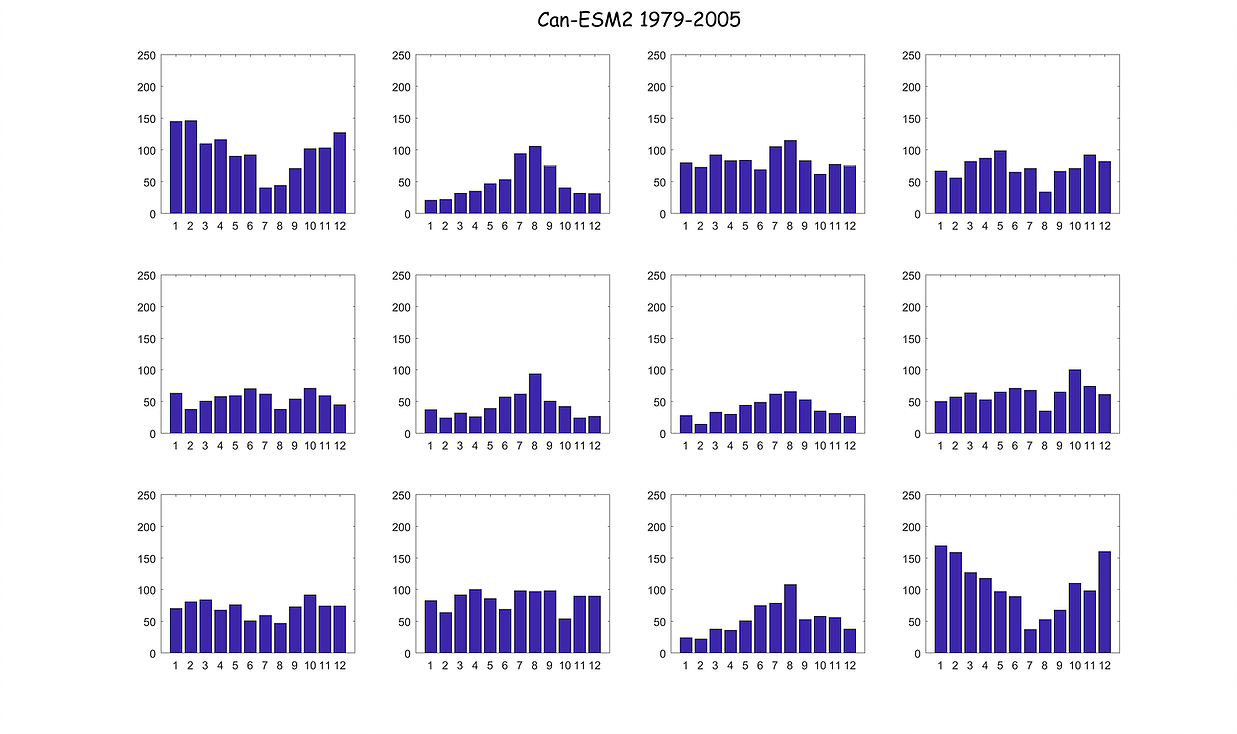

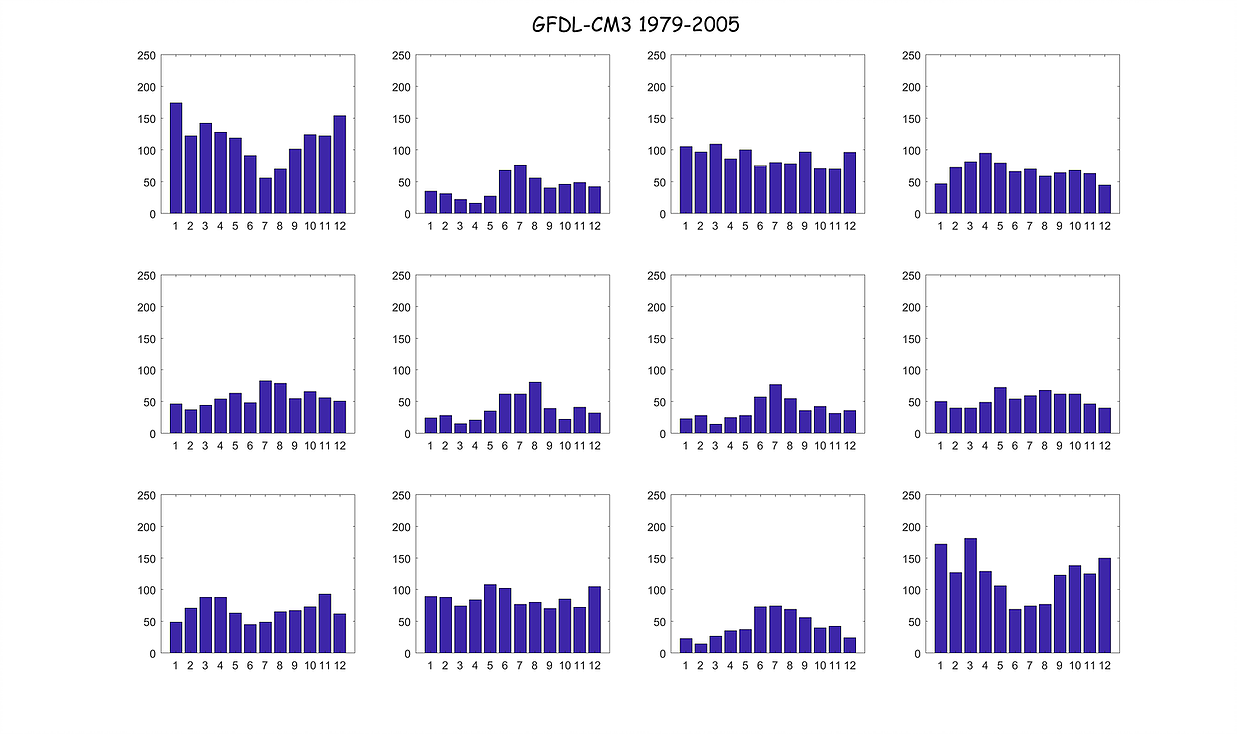


**Figure S2:** Monthly distributions of nodes in model simulations. Monthly distribution of node frequency during 1979-2005 for (a) NCEP/NCAR reanalysis output and historical simulations from (b) CCSM4, (c) Can-ESM2, and (d) GFDL-CM3. The vertical axis is number of days/month, horizontal axis is month of the year.


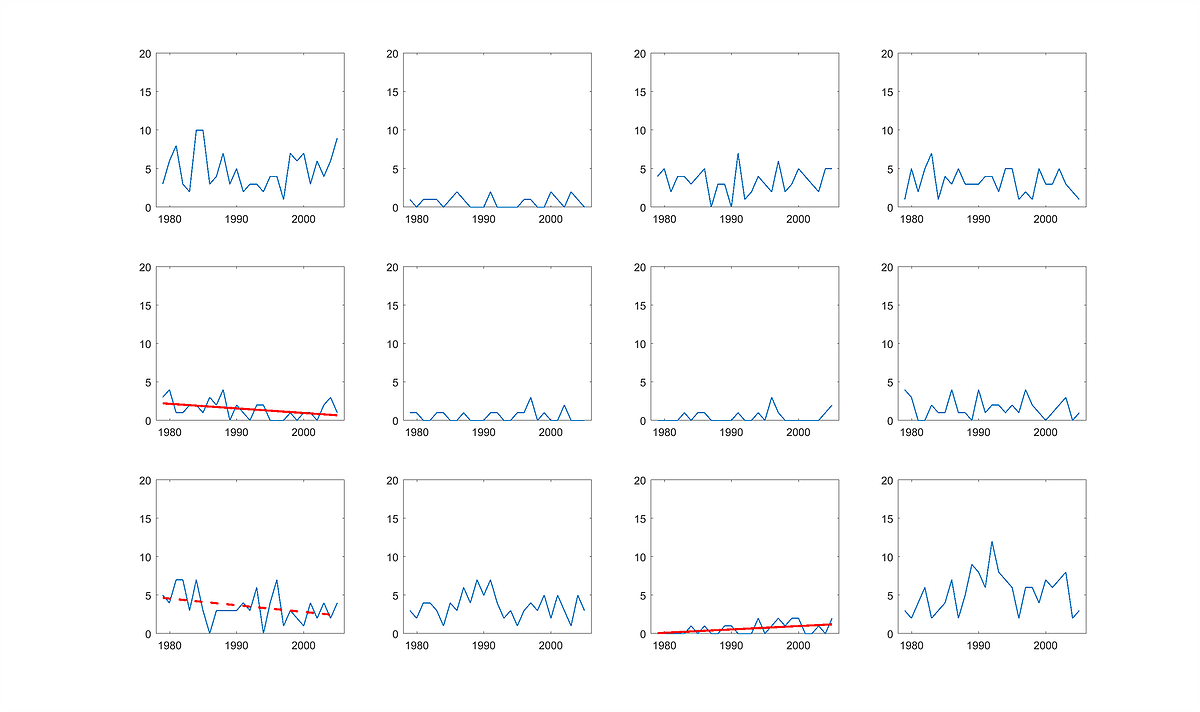

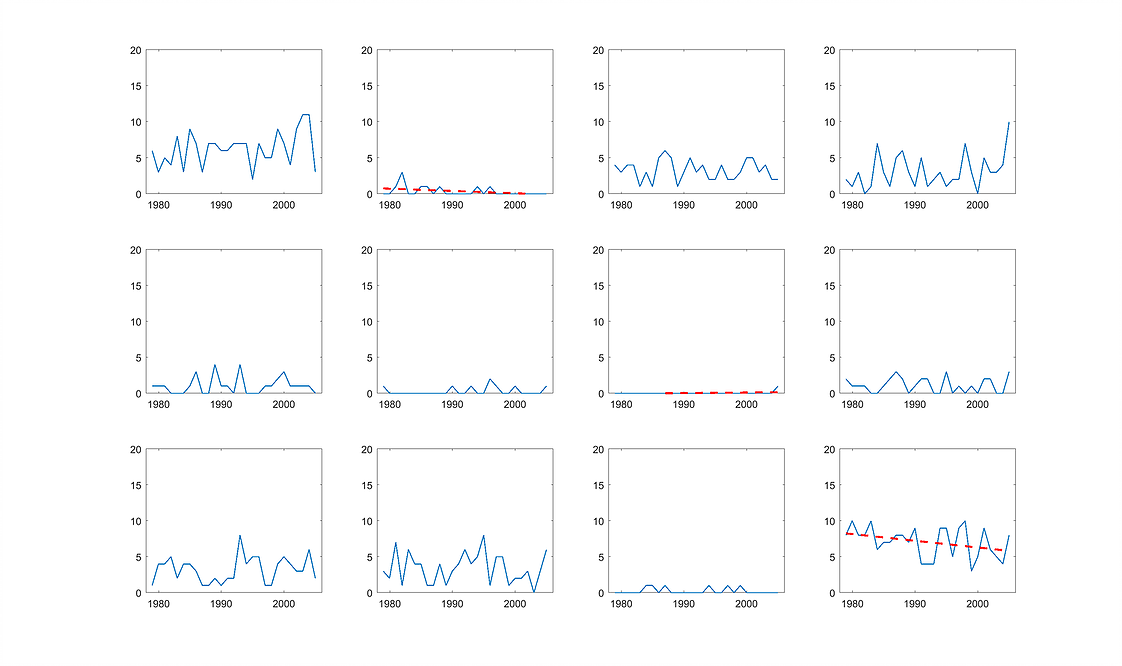


**d**

**c**

**b**

**a**


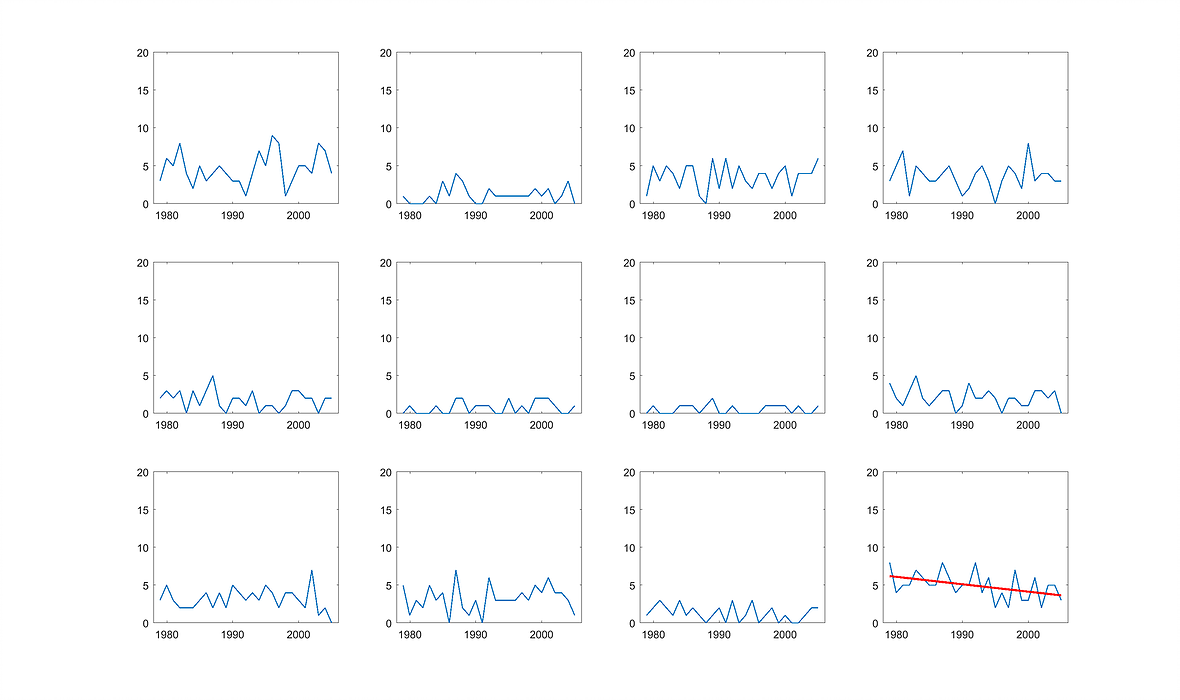

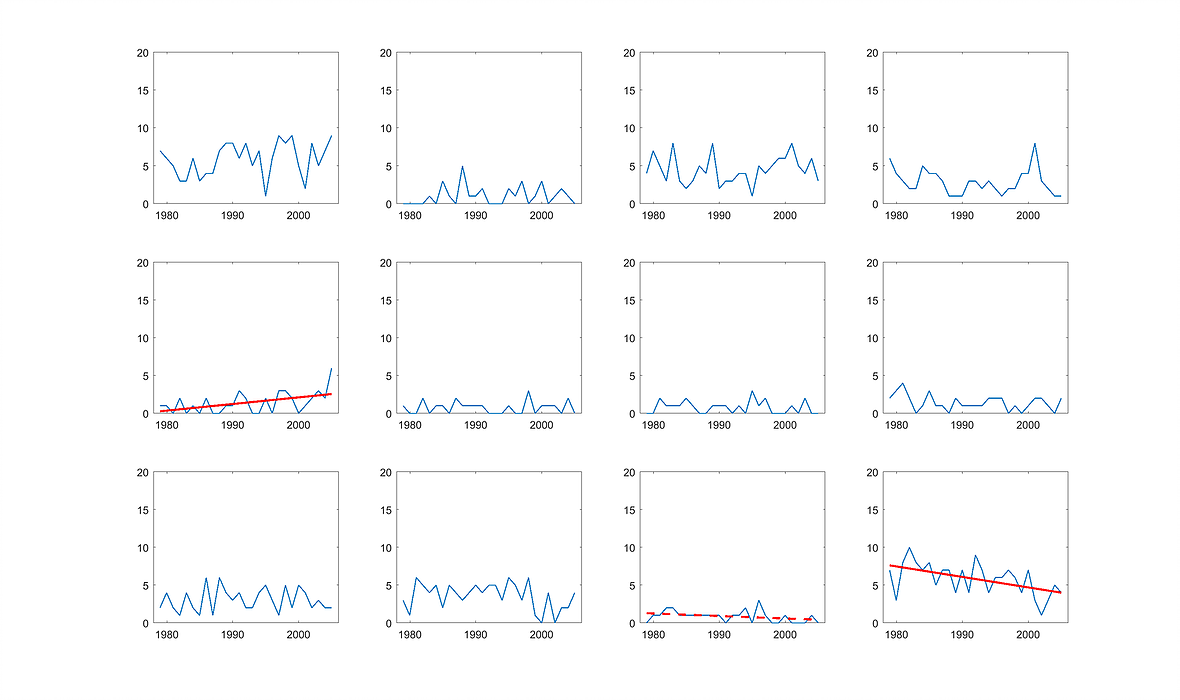


**Figure S3:** LDEs/year in historical simulations. Time series of LDEs/year during 1979-2005 for (a) NCEP/NCAR reanalysis output and historical simulations from (b) CCSM4, (c) Can-ESM2, and (d) GFDL-CM3. The vertical axis is number of LDEs/year.


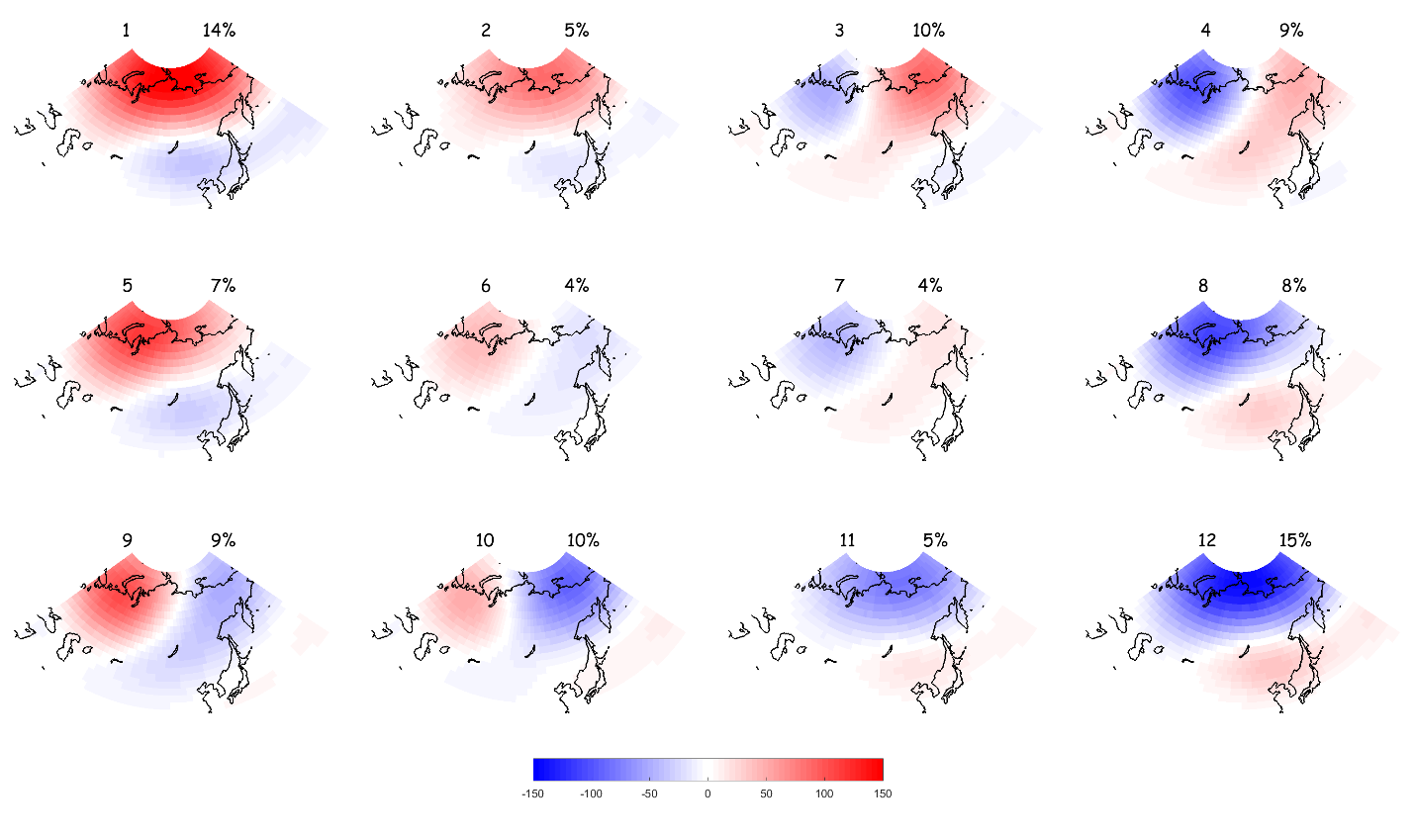


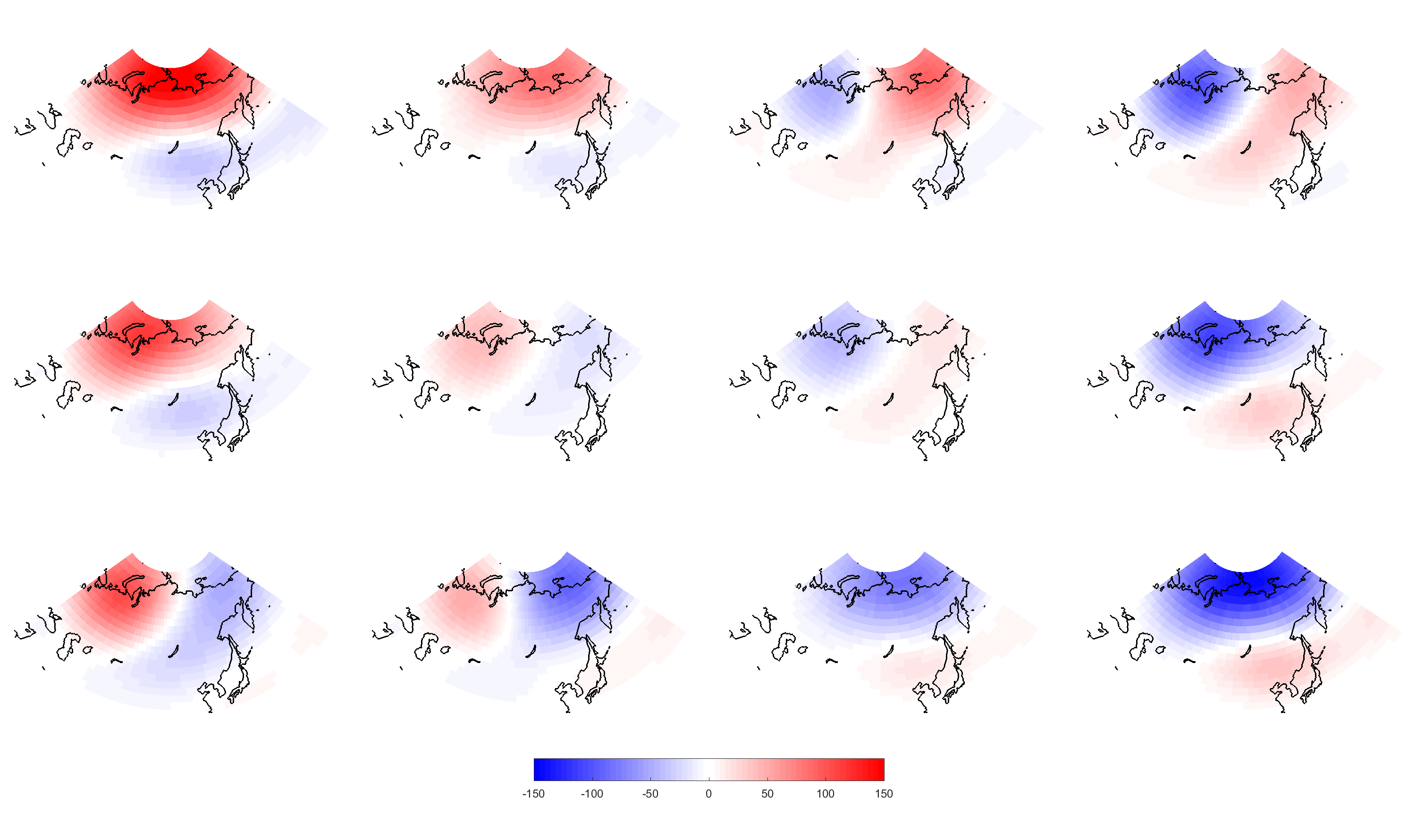


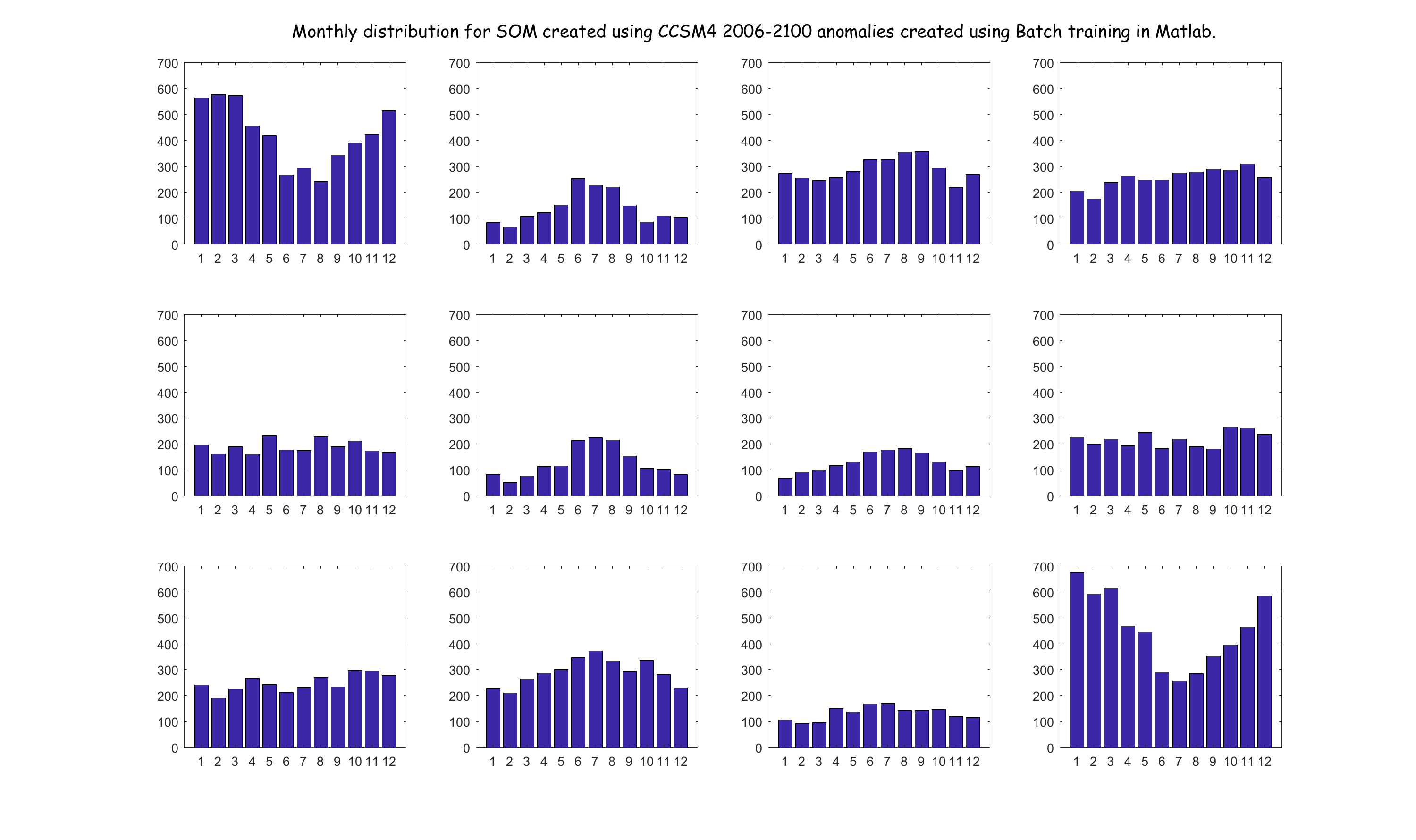


**Figure S4:** Projected patterns and distributions. (top) SOM matrix created using simulated daily 500 hPa anomaly fields (m) from CCSM4 spanning 2006 to 2100. Node number is indicated above and left of each pattern, and its relative frequency (% of total days) is indicated in upper right. (bottom) Monthly distribution of days in each SOM node. Vertical axis is number of days, horizontal axis is month.


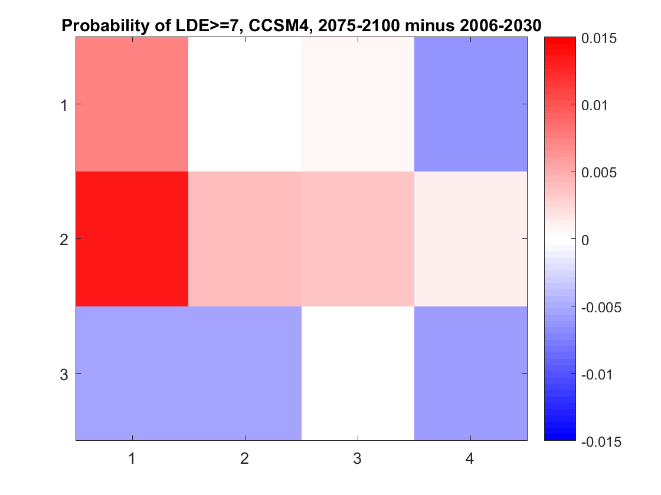

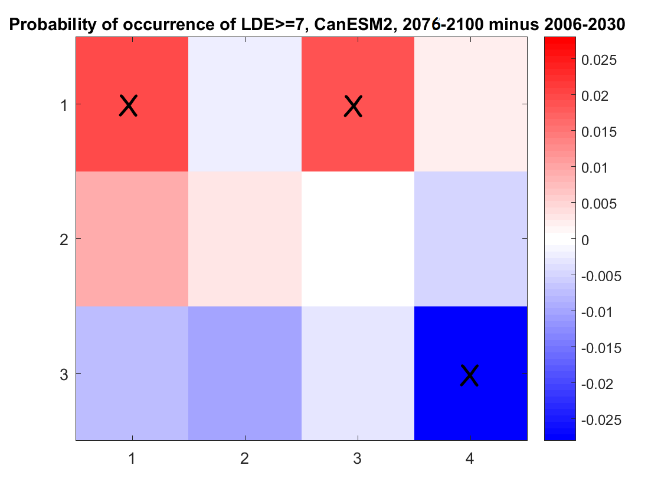


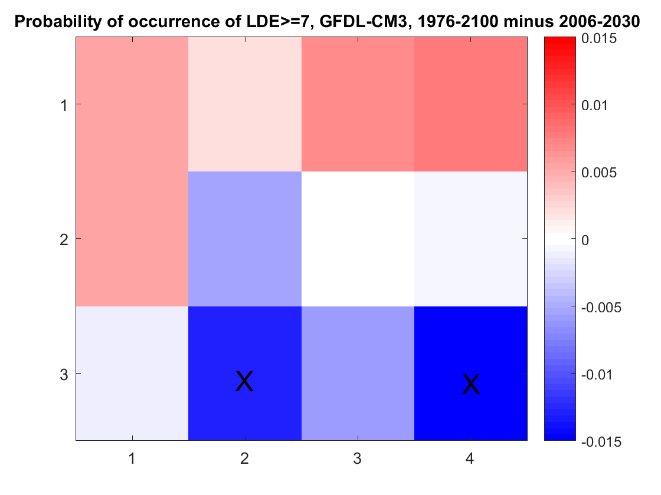


**Figure S5:** As in Fig. 7 but for projections by (a) CCSM4, (b) Can-ESM2, and (c) GFDL-CM3 under RCP 8.5 forcing, 2075 to 2100 minus 2006 to 2030. Differences significant with confidence exceeding 90% are indicated with an X.
